# Allocation Variable-Based Probabilistic Algorithm to Deal with Label Switching Problem in Bayesian Mixture Models

**DOI:** 10.1371/journal.pone.0138899

**Published:** 2015-10-12

**Authors:** Jia-Chiun Pan, Chih-Min Liu, Hai-Gwo Hwu, Guan-Hua Huang

**Affiliations:** 1 Department of Mathematics, National Chung Cheng University, Chiayi, Taiwan; 2 Department of Psychiatry, National Taiwan University Hospital and National Taiwan University College of Medicine, Taipei, Taiwan; 3 Institute of Statistics, National Chiao Tung University, Hsinchu, Taiwan; Feng Chia University, TAIWAN

## Abstract

The label switching problem occurs as a result of the nonidentifiability of posterior distribution over various permutations of component labels when using Bayesian approach to estimate parameters in mixture models. In the cases where the number of components is fixed and known, we propose a relabelling algorithm, an allocation variable-based (denoted by AVP) probabilistic relabelling approach, to deal with label switching problem. We establish a model for the posterior distribution of allocation variables with label switching phenomenon. The AVP algorithm stochastically relabel the posterior samples according to the posterior probabilities of the established model. Some existing deterministic and other probabilistic algorithms are compared with AVP algorithm in simulation studies, and the success of the proposed approach is demonstrated in simulation studies and a real dataset.

## Introduction

Finite mixture models provide a flexible way to model heterogeneous data, and have been applied to a wide variety of data in social, medical and physical science. Overviews of applications of finite mixture models can be found in Titterington et al. [1] and McLachlan and Peel [2].

The likelihood function of the finite mixture model is invariant when switching component labels. In the last decades, the development of Markov chain Monte Carlo (MCMC) methods [3] and progress of computer technology facilitate the popularity of performing Bayesian analysis for finite mixture models. In the Bayesian setting, if the prior information does not distinguish the components of the mixture model, the resulting posterior distributions will be invariant to all permutations of component labels. Hence, the ergodic averages over the MCMC samples from the posterior distributions are meaningless. This is termed as the *label switching problem* [4, 5].

Many approaches have been proposed to deal with the label switching problem in Bayesian analysis. The most commonly used approach is to impose some artificial ordering constraints on model parameters (OC algorithm) [6, 7]. However, the poor choice for the constrained parameters may not provide a satisfactory solution [4, 7]. Celeux et al. [8] and Stephens [5] proposed the decision theoretic approach that minimizes a selected Monte Carlo risk. Stephens [5] (KL algorithm) suggested a particular choice of loss function based on the Kullback-Leibler divergence to measure the similarity of posterior allocation probabilities. Grün and Leisch [9] developed a more flexible risk-based algorithm to deal with more practical situations in real-world applications. These algorithms designed to minimize Monte Carlo risk can be regarded as imposing a sophisticated constraint through a loss function.

Other relabelling approaches require more sophisticated algorithms. Papastamoulis and Iliopoulos [10] used equivalence classes representatives (ECR algorithm) to reduce symmetric posterior distribution to nonsymmetric ones, which can be used to deal with the label switching problem. Yao and Lindsay [11] (HPD algorithm) used each MCMC sample as the starting point in an ascending algorithm, and labeled the sample based on the posterior mode to which the algorithm converged. Sperrin et al. [12] who proposed the probabilistic relabelling methods (SJW algorithm) considered a probabilistic learning mechanism to avoid “over-correct” relabels. Rodriguez and Walker [13] proposed an iterative version of the ECR algorithm (the iterative version 2 of the ECR algorithm: ECR2 algorithm), which did not require a good pivot estimate from the start, but improved it via an iterative algorithm. In ECR2, the allocation probabilities needed to be stored. They also develop a deterministic relabelling algorithm that uses the relationship between the observed data and allocation variables to devise a *K*-means type of loss function (DBS algorithm).

In this paper, an allocation variable based probabilistic relabelling approach (AVP algorithm) is proposed to find the labelling functions. The proposed algorithm is developed under the assumption that the posterior distributions of allocation variables are independent. The AVP algorithm is compared with other six existing relabelling algorithms (KL, ECR, HPD, SJW, ECR2 and DBS) in simulation studies. In real data analysis, schizophrenia syndrome scale data fitted by latent class model is used to demonstrate that labels can be identified well by using the proposed algorithm.

## The Label Switching Phenomena

### Bayesian Analysis of Finite Mixture Models

A finite mixture model composed of *K* components is of the form

$$\begin{array}{c} p\left(y|\theta \right)=\sum_{k=1}^{K}\eta_{k}f\left(y|\phi_{k},\psi \right), \end{array}$$

where *y* is the random variable (vector) of response, *ϕ*_*k*_ is the component specific parameter of density *f*, *η*_*k*_ is the component weight with *η*_*k*_ > 0 and $\sum_{k=1}^{K}\eta_{k}=1$, *ψ* is the parameter common to all components, and *K* is considered as fixed and known in this paper. Here we denote *θ*_*k*_ = (*η*_*k*_, *ϕ*_*k*_), and ***θ*** = (*θ*_1_, …, *θ*_*K*_, *ψ*). The likelihood for ***θ*** is

$$\begin{array}{c} L(\theta |y)=\prod_{i=1}^{n}\{\eta_{1}f\left(y_{i}|\phi_{1},\psi \right)+\ldots +\eta_{K}f\left(y_{i}|\phi_{K},\psi \right)\}, \end{array}$$

where **y** = (*y*_1_, …, *y*_*n*_) are independent observations from a mixture density *p*(⋅∣***θ***).

### Data Augmentation

In Bayesian analysis of finite mixture models, one can add missing data perspective into models to interpret the data formulation [7]. This is done by augmenting the data with latent class membership random variable (called allocation variable in this paper) *C*_*i*_, *i* = 1, …, *n*, where *C*_*i*_ indicates the class membership of observation *y*_*i*_. If *C*_*i*_ = *k*, the observation *y*_*i*_ is regarded as drawn from the *k*th component density. Then, we can assume that data *y*_*i*_ given both *C*_*i*_ and ***θ*** has distribution

$$\begin{array}{c} y_{i}|\left(C_{i}=k,\theta \right)∼f\left(y_{i}|\phi_{k},\psi \right), \end{array}$$

and *p*(*C*_*i*_ = *k*∣***θ***) = *η*_*k*_. The use of data augmentation technique simplifies the expression of likelihood; therefore, facilitate the MCMC simulation for posterior distributions.

Under a Bayesian framework, we specify prior distribution *p*(***θ***) for parameters ***θ***. The joint posterior distribution of ***θ*** and **C** are proportional to *L*(***θ***, **C**∣**y**) × *p*(***θ***), where **C** = (*C*_1_, …, *C*_*n*_) and $L(\theta ,C|y)=\overset{n}{\underset{i=1}{\prod }}\left\{\eta_{\;C_{i}}f(y_{i}|\phi_{C_{i}},\psi )\right\}$. The drawing of one parameter is full conditional on the other parameters. The procedures to draw the posterior samples of each element of ***θ*** and **C** are listed as follows:

**Step 1:** Update the component weights *η*_*k*_, for *k* = 1, …, *K*;
**Step 2:** Update the component specific parameter *ϕ*_*k*_, for *k* = 1, …, *K*;
**Step 3:** Update the common parameter *ψ*;
**Step 4:** Update the allocation variable *C*_*i*_, for *i* = 1, …, *n*.

Step 1 is usually completed by giving a Dirichlet prior distribution *D*(*e*_1_, …, *e*_*K*_) for (*η*_1_, …, *η*_*K*_), where *e*_*k*_’s are the hyperparameters. Given on the values of **C**, *ϕ*_1_, …, *ϕ*_*K*_ and *ψ*, the full conditional distribution of (*η*_1_, …, *η*_*K*_) is *D*(*e*_1_+*n*_1_, …, *e*_*K*_+*n*_*K*_), where $n_{k}=\sum_{i=1}^{n}I\left\{C_{i}=k\right\}$. Given the values of **C** and *η*_1_, …, *η*_*K*_, Step 2 and Step 3 are standard steps for MCMC simulation and the way to simulate samples is model-dependent. Further blocking of ***θ*** is possible necessary for convenient sampling in each block. Examples of simulating ***θ*** are illustrating in Sections simulation studies and real data analysis. Given the values of ***θ***, the implementation of Step 4 is carried out by drawing *C*_*i*_ from a multinomial distribution with parameters *π*_*i*1_(***θ***), …, *π*_*iK*_(***θ***), where

$$\begin{array}{c} \pi_{ik}\left(\theta \right)=\frac{\eta_{k}f\left(y_{i}|\phi_{k},\psi \right)}{\sum_{j=1}^{K}\eta_{j}f\left(y_{i}|\phi_{j},\psi \right)}. \end{array}$$ (1)

Allocation variable *C*_*i*_ can be expressed as a set of binary random variables as well. Define a set of binary random vector (*S*_*i*1_, …, *S*_*iK*_), and let *S*_*im*_ = 1 if *C*_*i*_ = *m* and *S*_*ik*_ = 0 for all *k* ≠ *m*. The allocation variable **C** forms an *n* × *K* allocation variable matrix **S** = [*S*_*ik*_]_1 ≤ *i* ≤ *n*, 1 ≤ *k* ≤ *K*_ that summaries the allocation informations of **C**.

### The Label Switching Phenomenon

There are *K*! possible permutations of {1, …, *K*}. Let *v* ^*q*^ be the *q*th permutation among the *K*! possible permutations. The permutation function *v* ^*q*^ transfers the original index {1, …, *K*} to {*v* ^*q*^(1), …, *v* ^*q*^(*K*)}. Define the *q*th corresponding permutation of the parameter ***θ*** by

$$\begin{array}{c} v^{q}\left(\theta \right)=\left(\theta_{v^{q}\left(1\right)},\ldots ,\theta_{v^{q}\left(K\right)},\psi \right), \end{array}$$

and of allocation variable matrix **S** by *v* ^*q*^(**S**) = [*S*_*iv*^*q*^(*k*)_]_1 ≤ *i* ≤ *n*, 1 ≤ *k* ≤ *K*_. The label switching problem arises when likelihood *L*(***θ***∣**y**) is permutation invariant, *L*(***θ***∣**y**) = *L*(*v* ^*q*^(***θ***)∣**y**) for all *q* = 1, …, *K*!. If the prior distributions of ***θ*** are also permutation invariant, the posterior distribution will also be invariant to any permutation function on parameters. Samples generated from MCMC are the simulation outputs of the permutation invariant likelihood and priors with unknown value of *q*; therefore, when Markov chain is stationary, every sample in MCMC simulation is a sample from permutation invariant posterior distributions. Then the statistics, such as credible interval and posterior mean, inferred from the marginal posterior distributions become meaningless unless the inverse permutation function of every sample is discovered to relabel the MCMC outputs of ***θ***.

Although the label switching phenomenon causes difficulty in inferences of the posterior distributions, the phenomenon can help generate a useful convergence diagnostics of MCMC simulation jasra markov 2005. A Markov chain that fails to visit all permutation states with approximately equal frequency can be viewed as a warning message of nonstationarity. Hence, for ensuring a Markov chain to reach its stationary state, Frühwirth-Schnatter [15] proposed a dynamic switching procedure, called *permutation sampler*, for Bayesian mixture models to force the Markov chain quickly exploring all possible permutation states. This indicates that label switching phenomenon is a desired property. Therefore, the posterior distribution of parameters is a mixture of *K*!-component densities. Frühwirth-Schnatter [15] termed samples that visited all permutation states with approximately equal frequency as *unconstrained samples*. A formal proof given by Papastamoulis and Iliopoulos [16] states that the permutation sampler converges at least as fast as the unconstrained sampler. In the following, we adopt Frühwirth-Schnatter’s procedure and inherit their terminologies.

## Proposed Relabelling Method

The permutation function that has worked on ***θ*** and **S** is arbitrary and not observed. We treat the unobservable index of the permutation function as a latent random variable *τ* taking one value of {1, …, *K*!} and $\mathrm{Pr}[\tau =k]=\frac{1}{K!}$ for *k* = 1, …, *K*! fruhwirth-schnatter markov 2001. Another random variable *σ* is the index of the inverse permutation function of *τ*, where ***θ*** = *v* ^*τ*^(*v* ^*σ*^(***θ***)) = *v* ^*σ*^(*v* ^*τ*^(***θ***)) and **S** = *v* ^*τ*^(*v* ^*σ*^(**S**)) = *v* ^*σ*^(*v* ^*τ*^(**S**)). If the value of *τ* is observed, the inverse permutation function *v* ^*σ*^ is known and can transfer ***θ*** and **S** back to the one of the *K*! permuted posterior densities of the unconstrained samples.

In subsequent sections, the Markov chain is assumed to be stationary and ergodic. For MCMC samples {(***θ*** ^*t*^, **S** ^*t*^):*t* = 1, …}, let *τ* ^*t*^ be the latent random variable of the unobserved permutation function at time *t*, and let *σ* ^*t*^ be the index of its corresponding inverse permutation function.

We propose an allocation variable based probabilistic (AVP) relabelling algorithm to deal with label switching problem. The AVP algorithm can be regarded as being developed under the assumption where the posterior distributions of the allocation random variables *C*_1_, …, *C*_*n*_ are independent. The independence assumption in the posterior distribution (*C*_1_, …, *C*_*n*_)∣**y** usually does not hold because of the variability from prior distribution *p*(***θ***). We have imposed such an independence assumption to obtain a tractably practical solution to label switching phenomenon in Bayesian mixture models. Similar simplifications were assumed to other Bayesian techniques, such as variational Bayes approaches (see e.g., Corduneanu and Bishop, 2001 [17]; Bishop, 2006 [18]). In the rest of this section, we assume that the posterior distributions of *C*_1_, …, *C*_*n*_ are independent, and ***π***_0_ = [*π*_0, *ik*_]_1 ≤ *i* ≤ *n*, 1 ≤ *k* ≤ *K*_ denotes the parameters of the posterior distribution of **S**.

Each posterior sample **S** is the consequence of label switching with an unknown permutation. The model of **S** can be constructed according to an unknown permutation random variable *τ* (or the relabelling random variable *σ*) and the parameters *π*_0_. We use multinomial distribution to model allocation variables (*S*_*i*1_, …, *S*_*iK*_) with *S*_*ik*_ taking value on 0 or 1 for all *k* and $\sum_{k=1}^{K}S_{ik}=1$. Then the probability mass function of (*S*_*i*1_, …, *S*_*iK*_) is $\prod_{k=1}^{K}\pi_{0,iv^{q}\left(k\right)}^{S_{ik}}$. Since the allocation variables are assumed to be independent, the posterior probability density at realized sample point **s** given **y** and *τ* = *q* could be modeled by

$$\begin{array}{c} \mathrm{Pr}\left[S=s|y,\tau =q\right]=\prod_{i=1}^{n}\prod_{k=1}^{K}\pi_{0,iv^{q}\left(k\right)}^{s_{ik}}. \end{array}$$

Let the probability Pr[*τ* = *q*∣**y**] be denoted by *w*_*q*_. Then the posterior probability density of **S** at **s** is

$$\begin{array}{ccc} \mathrm{Pr}[S=s|y] & = & \sum_{q=1}^{K!}w_{q}\mathrm{Pr}\left[S=s|y,\tau =q\right]=\sum_{q=1}^{K!}w_{q}\prod_{i=1}^{n}\prod_{k=1}^{K}\pi_{0,iv^{q}\left(k\right)}^{s_{ik}}. \end{array}$$ (2)

The value of *w*_*q*_ is the proportion of the value *q* occurred in the random variable *τ* in the Markov chain. When the Markov chains is stationary, relative frequency of samples generated from different sample points of *τ* will be eventually close, and hence the proportion of the different values of *τ* should be equal. This means if *T* is sufficiently large, the chains will achieve $w_{1}=\ldots =w_{K!}=\frac{1}{K!}$ fruhwirth-schnatter markov 2001. In the label switching problem, relabelling random variable *σ* is of our interest. We can rewrite Eq (2) through random variable *σ* as

$$\begin{array}{ccc} \mathrm{Pr}[S=s|y] & = & \sum_{m=1}^{K!}w_{m}\mathrm{Pr}\left[S=s|y,\sigma =m\right]=\sum_{m=1}^{K!}\frac{1}{K!}\prod_{i=1}^{n}\prod_{k=1}^{K}\pi_{0,ik}^{s_{iv^{m}\left(k\right)}}, \end{array}$$ (3)

where *v* ^*m*^ is the inverse permutation function of *v* ^*q*^ such that $v^{m}\left(v^{q}\left(S\right)\right)=S$.

To estimate parameters *π*_0_ in model (3), let $L_{ij}^{t}=\sum_{k=1}^{K}S_{ik}^{t}S_{jk}^{t}$ and

$$\begin{array}{c} f_{t}\left(i,j\right)=L_{ij}^{t}-\sum_{k=1}^{K}\pi_{0,ik}\pi_{0,jk} \end{array}$$ (4)

with restriction $\pi_{0,lK}=1-\sum_{k=1}^{K-1}\pi_{0,lk},l=i,j$. Let

$$\begin{array}{c} g_{T}\left(i,j\right)=\frac{\sum_{t=1}^{T}f_{t}\left(i,j\right)}{T}={\bar{L}}_{ij}-\sum_{k=1}^{K}\pi_{0,ik}\pi_{0,jk}. \end{array}$$ (5)

where ${\bar{L}}_{ij}=\frac{\sum_{t=1}^{T}L_{ij}^{t}}{T}$. Notice that the expectation of Eq (5) is 0 when *C*_*i*_ and *C*_*j*_ are independent for all *i*, *j* and *i* ≠ *j*. Then *E*(∑_*i* ≠ *j*_ *g*_*T*_(*i*, *j*)) = 0 is a moment equation for *π*_0_. According to this equation, an object function is defined as

$$O\left(\pi_{0}\right)=\frac{\overset{n-1}{\underset{i=1}{\sum }}\overset{n}{\underset{j=i+1}{\sum }}{\left(g_{T}\left(i,j\right)\right)}^{2}}{n(n-1)/2}.$$ (6)

Notice that Eq (4) depending on {*π*_0, *i*1_, …,*π*_0, *jK*_} is invariant to different label permutations, and so do Eqs (5) and (6). The minimizer with respect to *π*_0_ in Eq (6), ${\hat{\pi }}_{0}$, obtained through Newton’s method is the Generalized Method of Moments (GMM) estimator. The GMM estimator ${\hat{\pi }}_{0}$ has been found to have several large sample properties in Hansen [19], including that ${\hat{\pi }}_{0}$ approximates *π*_0_ almost surely.

To estimate the value of *σ* at different time point, let *σ* ^*t*^ denote the random variable *σ* at time *t*. The estimation of *σ* ^*t*^ can be obtained through the following posterior probability:

$$\begin{array}{ccc} \xi_{m}^{t} & := & \mathrm{Pr}[\sigma^{t}=m|S^{t}=s,y]\\ & = & \frac{\mathrm{Pr}\left[S^{t}=s|y,\sigma^{t}=m\right]\mathrm{Pr}\left[\sigma^{t}=m|y\right]}{\sum_{l=1}^{K!}\mathrm{Pr}\left[S^{t}=s|y,\sigma^{t}=l\right]\mathrm{Pr}\left[\sigma^{t}=l|y\right]}\\ & = & \frac{\prod_{i=1}^{n}\prod_{k=1}^{K}{\left[\pi_{0,ik}\right]}^{s_{iv^{m}\left(k\right)}}}{\sum_{l=1}^{K!}\prod_{i=1}^{n}\prod_{k=1}^{K}{\left[\pi_{0,ik}\right]}^{s_{iv^{l}\left(k\right)}}}. \end{array}$$ (7)

Based on these posterior probabilities, we adopt the following stochastic algorithm (termed AVP algorithm) to estimate *σ* ^*t*^, for each *t* = 1, …, *T*.

**AVP Algorithm.**

**Step A:** Numerically solve the GMM estimator ${\hat{\pi }}_{0}$ from Eq (6).
**Step B:** For *t* = 1, …, *T*, estimate $\xi_{m}^{t}$ by substituting GMM parameter estimates, ${\hat{\pi }}_{0,ik}$’s, into Eq (7) to obtain ${\hat{\xi }}_{m}^{t}$, *m* = 1, …, *K*!.
**Step C:** Randomly assign the relabelling permutation index at time *t*, ${\hat{\sigma }}^{t}$, to a value of {1, …, *K*!}, with probability $\left\{{\hat{\xi }}_{1}^{t},\ldots ,{\hat{\xi }}_{K!}^{t}\right\}$.

The AVP algorithm offers an approach that estimates the index of inverse permutation function. Then apply the estimate of permutation function $v^{{\hat{\sigma }}^{t}}$ to ***θ*** ^*t*^ for relabelling parameters. For the examples in simulation studies and real data application, the AVP algorithm is able to have satisfactory relabelled results.

## Simulation Studies

In this section, we compare the proposed AVP algorithm with various relabelling algorithms. First, we compare AVP with algorithms KL, ECR, SJW and HPD in poisson mixture models with fixed and known component weights and *K* = 2. With known component weights, we can then analytically show how these methods transform posterior distributions. Second, we compare AVP with more recent solutions ECR2 and DBS under normal mixture models with both known and unknown component weights. The comparison of AVP and ECR2 are studied under univariate normal mixture models with *K* = 3, and the comparison of AVP and DBS are studied under multivariate normal mixture models with *K* = 4. Except for the HPD and AVP algorithms, all the comparative algorithms are available to the label.switching package [20] of R software. Finally, the computation time of various relabelling algorithms for these simulation studies are summarized at the end of this section.

### Poisson Mixture Models with Known Component Weights

Poisson mixture models are studies in this section, and five relabelling methods are compared, including KL, ECR, HPD, SJW and AVP.

This simulation study generates data from a two-component poisson mixture model whose probability density function is

$$\begin{array}{c} f\left(y_{i}|\eta ,\phi \right)=\eta_{1}f\left(y_{i}|\phi_{1}\right)+\eta_{2}f\left(y_{i}|\phi_{2}\right), \end{array}$$ (8)

where ***η*** = (*η*_1_, *η*_2_), ***ϕ*** = (*ϕ*_1_, *ϕ*_2_), and *f*(*y*_*i*_∣*ϕ*_*k*_) is a poisson distribution with the parameter *ϕ*_*k*_ for the response *y*_*i*_. Simulate **y** = (y_1_, …,y_n_) under four scenarios: (1) *η*_1_ = *η*_2_ = 0.5, *ϕ*_1_ = 5, *ϕ*_2_ = 7 and *n* = 10; (2) *η*_1_ = *η*_2_ = 0.5, *ϕ*_1_ = 5, *ϕ*_2_ = 7 and *n* = 100; (3) *η*_1_ = 0.3, *η*_2_ = 0.7, *ϕ*_1_ = 5, *ϕ*_2_ = 5.5 and *n* = 10; and (4) *η*_1_ = 0.3, *η*_2_ = 0.7, *ϕ*_1_ = 5, *ϕ*_2_ = 5.5 and *n* = 100. In the following simulations, the component weights (i.e., *η*_1_ and *η*_2_) are treated as fixed and known values, and only the parameters in the component densities (i.e., *ϕ*_1_ and *ϕ*_2_) are of our interest and are estimated via MCMC simulation. Assume that priors for *ϕ*_1_ and *ϕ*_2_ are i.i.d. from the gamma distribution Γ(1.2, 0.2) with mean 6, and use the poisson-gamma model to obtain the posterior samples of ***ϕ***. While generating the posterior samples of ***ϕ***, set the values of ***η*** to be the true values under each scenario.

The Gibbs sampling scheme is adopted here to produce posterior samples {(***ϕ*** ^1^, **S** ^1^), …,(***ϕ*** ^*T*^, ***S*** ^*T*^)}, where the allocation variable matrix **S** ^*t*^ is an *n* × 2 matrix of which the element $S_{ik}^{t}$ is a 0/1 variable. $S_{ik}^{t}=1$ if the *i*th subject is attributed to the *k*th component in the *t*th MCMC iteration, and $S_{ik}^{t}=0$ otherwise. This sampling scheme starts with an initial value **S** ^0^, and runs for *t* = 1, …, *T* as follows:

**Step 1.** Generate $\phi_{k}^{t}$ from $\Gamma (1.2+\sum_{i=1}^{n}y_{i}S_{ik}^{t-1},0.2+\sum_{i=1}^{n}S_{ik}^{t-1})$ for *k* = 1, 2;
**Step 2.** Generate **S** ^*t*^ with its the element $S_{i1}^{t}$ from the Bernoulli distribution with probability $\frac{\eta_{1}{\left(\phi_{1}^{t}\right)}^{y_{i}}\exp\left\{\phi_{1}^{t}\right\}}{\sum_{k=1}^{2}\eta_{k}{\left(\phi_{k}^{t}\right)}^{y_{i}}\exp\left\{\phi_{k}^{t}\right\}}$ and set $S_{i2}^{t}=1-S_{i1}^{t}$ for *i* = 1, …, *n*, where *η*_1_ and *η*_2_ are fixed values and are therefore independent of *t*;
**Step 3.** Select the permutation sampler (1, 2) or (2, 1) with equal probability 0.5. If (1, 2) is chosen, the labels of components of (***θ*** ^*t*^, **S** ^*t*^) remain unchanged; else, alter the labels 1 and 2 of the components in (***θ*** ^*t*^, **S** ^*t*^), where $\theta^{t}=\left(\theta_{1}^{t},\theta_{2}^{t}\right)$, $\theta_{k}^{t}=\left(\eta_{k},\phi_{k}^{t}\right)$, *k* = 1, 2.

The permutation sampler applied in Step 3 has different functions for different scenarios. In Scenarios (1) and (2) where ***η*** values are fixed at *η*_1_ = *η*_2_ = 0.5, the Markov chain can produce label switching, and the permutation sampler is applied here to enhance quick convergence of MCMC and to obtain the unconstrained samples fruhwirth-schnatter markov 2001. In Scenarios (3) and (4) where ***η*** values are fixed at *η*_1_ = 0.3 and *η*_2_ = 0.7, the likelihood Eq (8) is not symmetric, and the usual Gibbs sampling without adopting Step 3 does not produce label switching. The permutation sampler used here can make the unconstrained posterior samples from likelihood

$$\begin{array}{c} 0.3f\left(y_{i}|\phi_{1}\right)+0.7f\left(y_{i}|\phi_{2}\right)\;\text{or}\;0.7f\left(y_{i}|\phi_{1}\right)+0.3f\left(y_{i}|\phi_{2}\right), \end{array}$$ (9)

which creates a “pseudo” label switching phenomenon. Then, we can apply various relabelling methods to the unconstrained samples of (*ϕ*_1_, *ϕ*_2_). The correctly labelled posterior samples of ***ϕ*** can be obtained by imposing an ordering constraint on ***η***. Hence, we can compare the relabelled results of algorithms with the correctly labelled posterior samples.

The Gibbs sampling scheme was run for 110,000 samples for each scenario. The first 10,000 samples were treated as the burn-in period, and the subsequent 100,000 samples were used for relabelling. Algorithms KL, ECR, HPD, SJW and AVP were applied to the unconstrained samples of each scenario.

**Fig 1:**
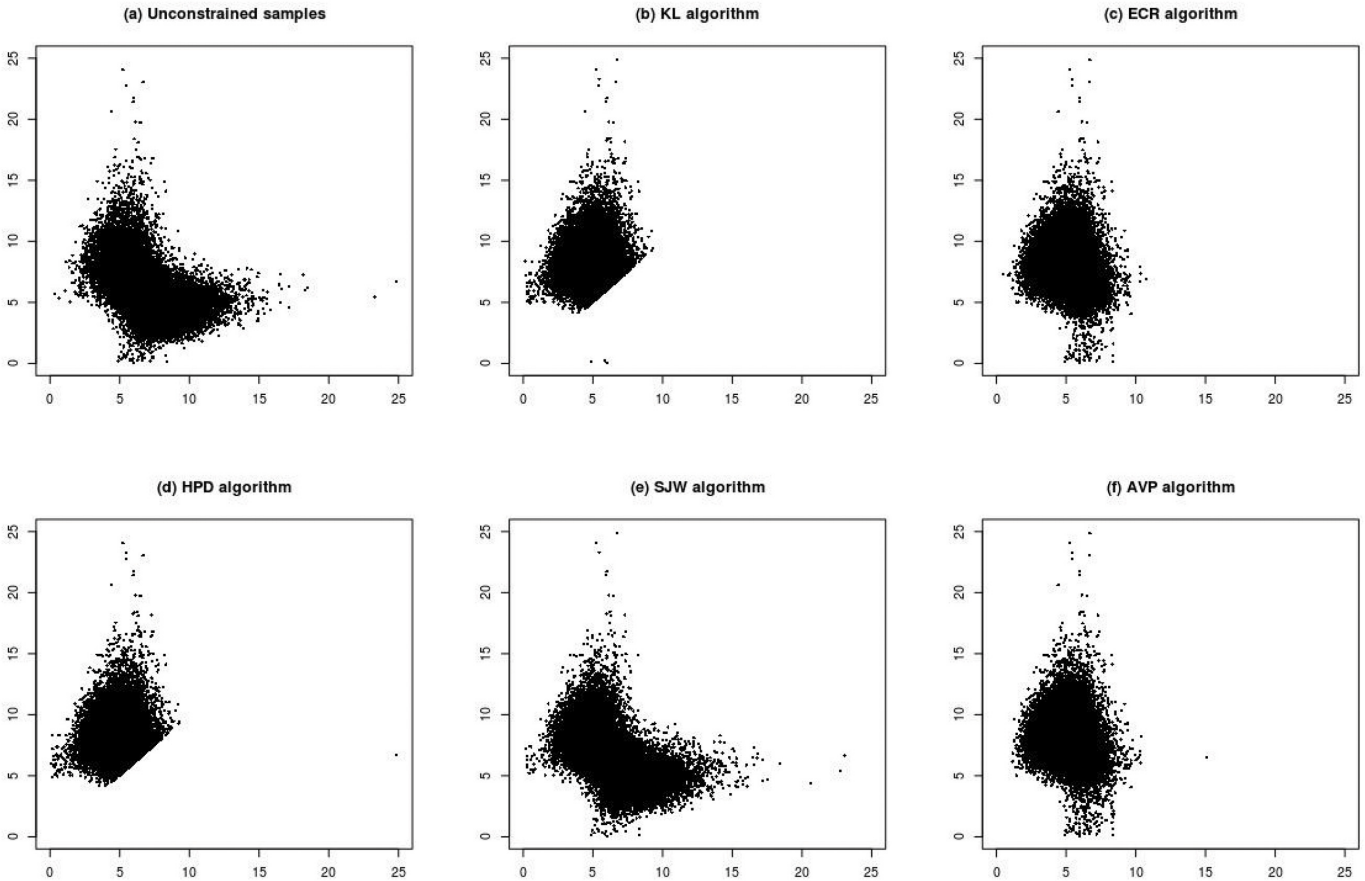
Plots (a)–(f) are scatter plots of posterior samples of (*ϕ*_1_, *ϕ*_2_) for Scenario (1) (n = 10, *ϕ*_1_ = 5, *ϕ*_2_ = 7, η_1_ = η_2_ = 0.5). Plot (a) is the unconstrained samples. Plots (b)–(f) are the relabelled samples under various relabelling algorithms.

Fig 1 shows the relabelled results under Scenario (1). Fig 1a shows a scatter plot of the unconstrained samples of ***ϕ***, which is symmetry along the 45 degree line. This means that the samples were explored well because of the use of permutation sampler. The Fig 1b–1f show the scatter plots of the relabelled results after adopting the five relabelling algorithms. Fig 1b and 1d show that KL and HPD assigned posterior samples of ***ϕ***’s lying below the 45 degree line to the other side. The results in these figures are almost the same as the ordinary constraint relabelling with the restriction *ϕ*_2_ ≥ *ϕ*_1_. Fig 1e shows that the results from the SJW algorithm are almost the same as those in Fig 1a, which does not seem to relabel the unconstrained samples well. The performance of the ECR algorithm shown in Fig 1c is almost the same as that of our AVP algorithm in Fig 1f.

**Fig 2:**
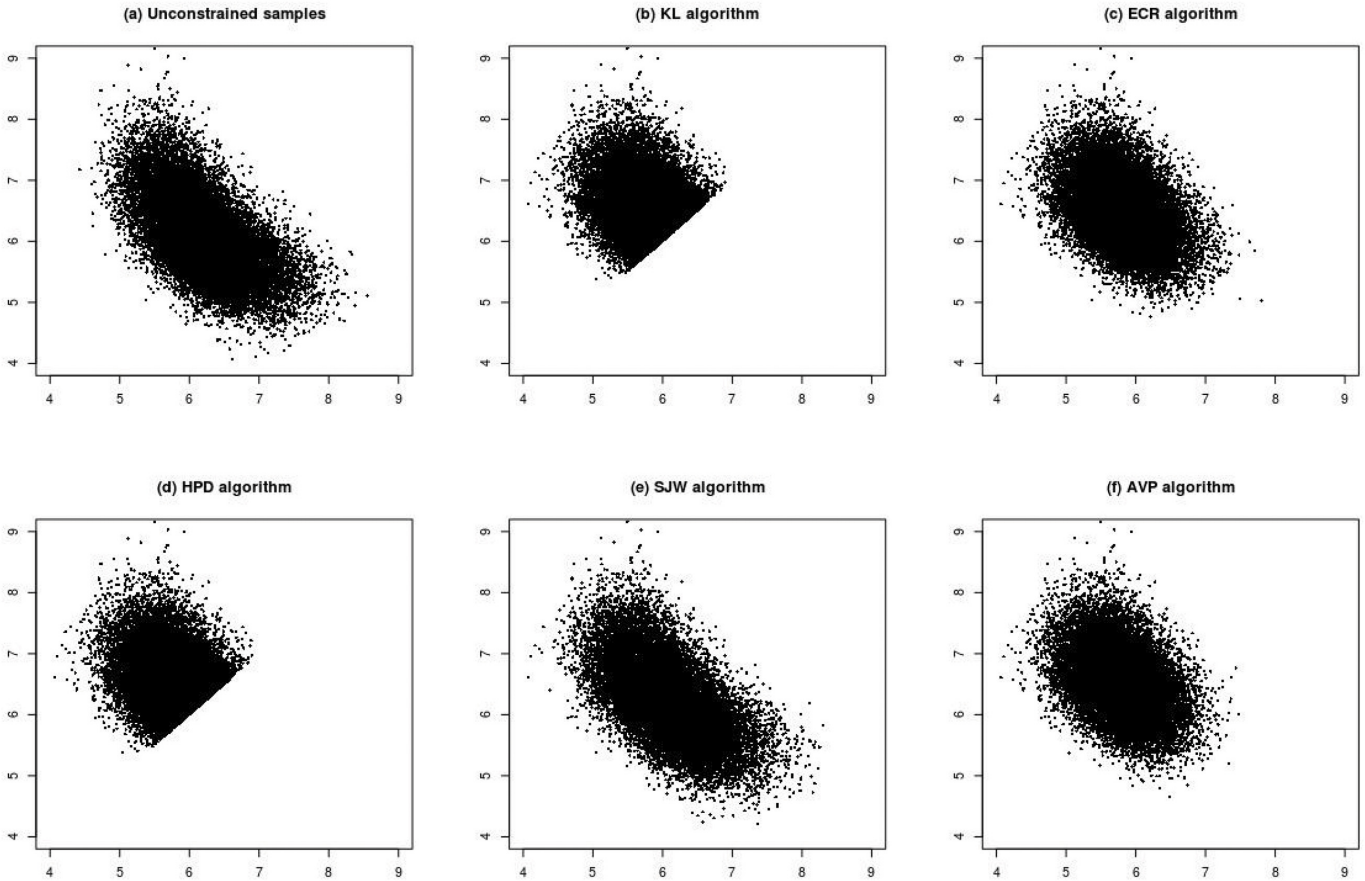
Plots (a)–(f) are scatter plots of posterior samples of (*ϕ*_1_, *ϕ*_2_) for Scenario (2) (n = 100, *ϕ*_1_ = 5, *ϕ*_2_ = 7, η_1_ = η_2_ = 0.5). Plot (a) is the unconstrained samples. Plots (b)–(f) are the relabelled samples under various relabelling algorithms.

To understand the effects of large samples, the sample size of Scenario (1) was increased from *n* = 10 to *n* = 100 (Scenario (2)). Fig 2 shows that the posterior samples are apparently more concentrated than those from *n* = 10. Conclusions from comparisons of KL, HPD and SWJ are consistent with those from *n* = 10. ECR (Fig 2c) and AVP (Fig 2f) have similar results, but it seems that ECR has posterior samples spreading more widely below the 45 degree line than AVP.

**Fig 3:**
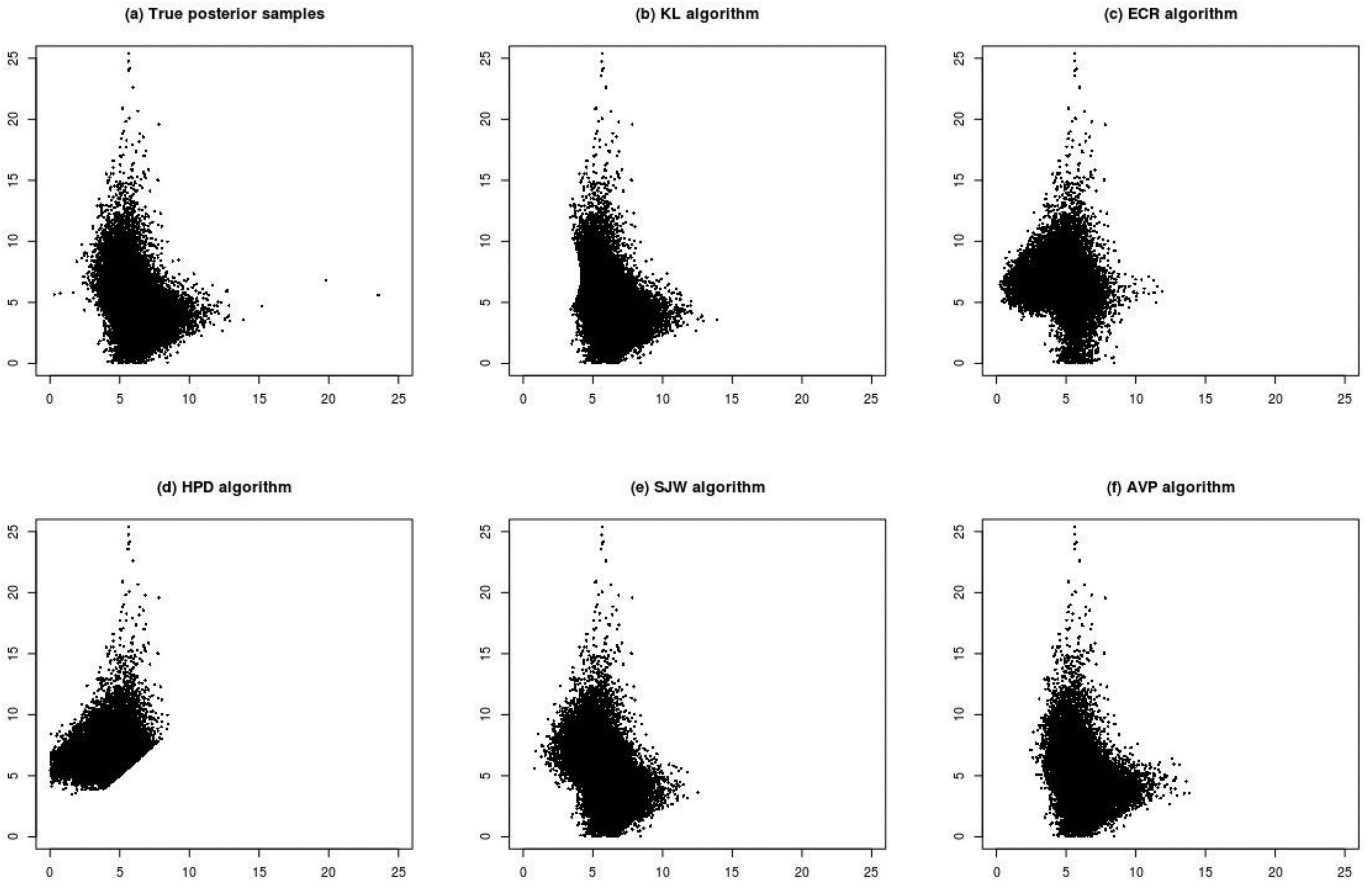
Plots (a)–(f) are scatter plots of posterior samples of (*ϕ*_1_, *ϕ*_2_) for Scenario (3) (n = 10, *ϕ*_1_ = 5, *ϕ*_2_ = 5.5, η_1_ = 0.3 and η_2_ = 0.7). Plot (a) is the posterior samples with correct labels. Plots (b)–(f) are the relabelled samples under various relabelling algorithms.

Fig 3 shows the results under Scenario (3). This scenario decreases the distance between *ϕ*_1_ and *ϕ*_2_, and allows the values of ***η*** to be unequal (*η*_1_ = 0.3 and *η*_2_ = 0.7). These settings place emphasis on the effect of the unequal weights and the reduced distance of ***ϕ***. Notice that, in Scenarios (3) and (4), ***η*** values are set to the fixed true values of *η*_1_ = 0.3 and *η*_2_ = 0.7. Therefore, the correctly labelled posterior distribution of ***ϕ*** can be obtained by restricting *η*_1_ < *η*_2_. Fig 3a presents a scatter plot of the correctly labelled posterior samples of ***ϕ***. The relabelled samples from HPD (Fig 3d) is the same to those of imposing an ordinary constraint *ϕ*_2_ ≥ *ϕ*_1_. The KL algorithm (Fig 3b) seems to move the relabelled sample points in the middle-left region to the opposite side symmetric to the 45 degree line. This phenomenon cannot be improved even if we use the correctly labelled posterior samples as initial points for the KL algorithm. Compared with the scatter plot of correctly relabelled posterior samples, AVP (Fig 3f) seems to generate the most similar results than ECR (Fig 3c) and SJW (Fig 3e) do.

**Fig 4:**
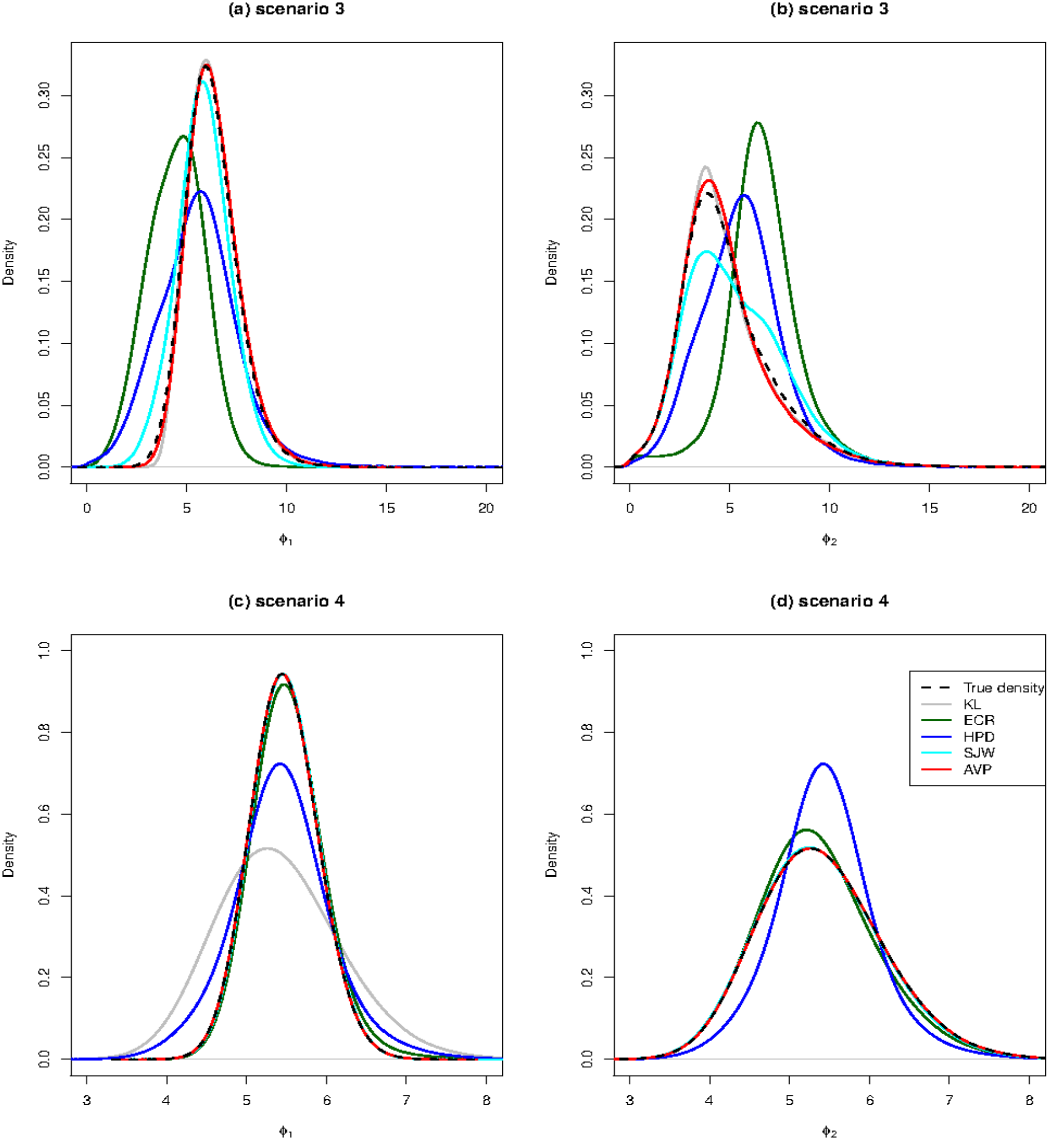
The density plots of relabelling samples from various relabelling methods in Scenarios (3) and (4). The black dashed line represents the density plot of the true posterior distributions. The grey, blue, purple, blue and red lines represent the density plots of KL, ECR, HPD, SJW and AVP, respectively. Plots (a) and (b) are the density plots of *ϕ*_1_ and *ϕ*_2_ for Scenario (3), respectively. Plots (c) and (d) are the density plots of *ϕ*_1_ and *ϕ*_2_ for Scenario (4), respectively.

Because the correctly labelled posterior samples are known in this scenario, the marginal distributions of ***ϕ*** of the relabelled samples from all relabelling methods can be compared with the true marginal densities, which are shown in Fig 4. Fig 4a and 4b show the density plots of *ϕ*_1_ and *ϕ*_2_ for Scenario (3), respectively. The density plot of the AVP algorithm nearly coincides with that of the correctly labelled posterior samples.

**Fig 5:**
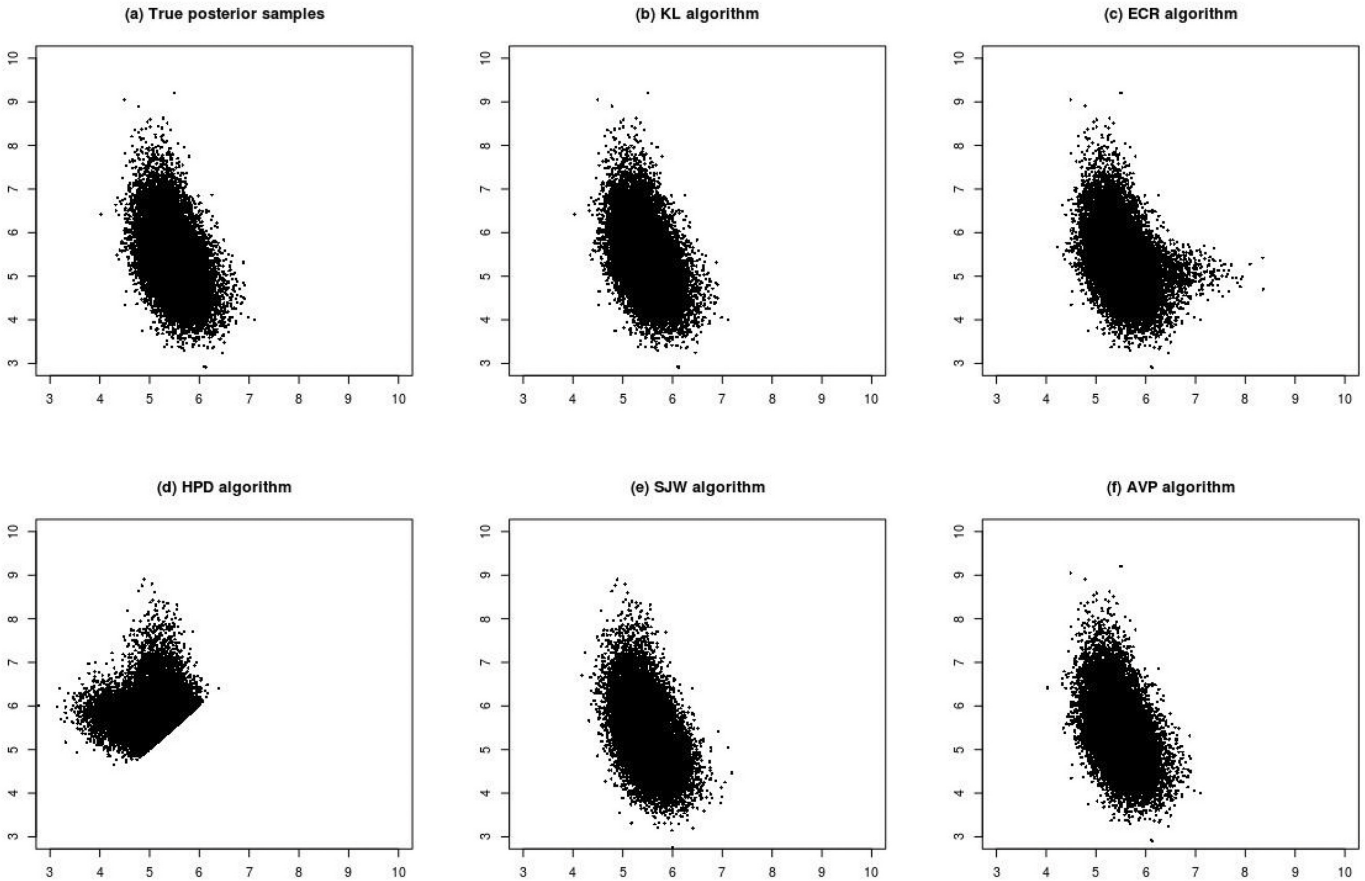
Plots (a)–(f) are scatter plots of posterior samples of (*ϕ*_1_, *ϕ*_2_) for Scenario (1) (n = 100, *ϕ*_1_ = 5, *ϕ*_2_ = 5.5, η_1_ = 0.3 and η_2_ = 0.7). Plot (a) is the posterior samples with correct labels. Plots (b)–(f) are the relabelled samples under various relabelling algorithms.

Fig 5 shows the results under Scenario (4), which increases the sample size of Scenario (3) to *n* = 100. In Scenario (4), the results from HPD (Fig 5d) are similar to those from the ordering constrainted samples. The performance of KL, SJW and AVP (Fig 5b, 5(e) and 5(f), respectively), is similar to that of the correctly labelled posterior samples (Fig 5a). ECR (Fig 5c) seems to gathers more sample points on the right side of the region. Fig 4c and 4(d) show the marginal density plots for Scenario (4). Except for HPD and ECR, other algorithms have density plots to coincide with that of correctly labelled posterior samples.

To produce a more reliable conclusion, simulated datasets are generated with 100 replications under Scenarios (1)–(4). Note that ***η*** are set to be fixed in these sencearios. The averages and standard deviations of posterior means over 100 replications are shown in Table 1.

**Table 1:** The Performance of AVP, ECR, SJW, HPD and KL in Poisson Mixture Models with Fixed Component Weights under Scenarios (1)–(4).

		Scenario (1)		Scenario (3)			
		*θ*_1_	*θ*_2_	*η*_1_	*η*_2_	*θ*_1_	*θ*_2_
OC	avg	5.885	6.665	0.300	0.700	4.761	5.856
	sd	0.803	1.123	0.000	0.000	0.782	1.006
AVP	avg	5.060	7.490	0.347	0.653	4.687	5.929
	sd	0.978	1.496	0.029	0.029	1.005	1.128
ECR	avg	5.068	7.483	0.364	0.636	4.985	5.632
	sd	0.905	1.447	0.051	0.051	0.999	1.158
SJW	avg	5.859	6.691	0.478	0.522	5.275	5.342
	sd	0.962	1.301	0.045	0.045	0.787	0.827
OC on ***θ***	avg	4.716	7.834	0.429	0.571	3.915	6.702
	sd	0.828	1.340	0.035	0.035	0.747	0.951
HPD	avg	4.719	7.831	0.429	0.571	3.916	6.700
	sd	0.830	1.341	0.035	0.035	0.746	0.951
KL	avg	4.722	7.828	0.320	0.680	4.572	6.045
	sd	0.831	1.341	0.036	0.036	0.969	1.116
		Scenario (2)		Scenario (4)			
		*θ*_1_	*θ*_2_	*η*_1_	*η*_2_	*θ*_1_	*θ*_2_
OC	avg	6.052	6.289	0.300	0.700	5.290	5.453
	sd	0.282	0.351	0.000	0.000	0.423	0.342
AVP	avg	5.248	7.094	0.300	0.700	5.290	5.453
	sd	0.366	0.543	0.000	0.000	0.425	0.343
ECR	avg	5.259	7.082	0.309	0.691	5.333	5.426
	sd	0.372	0.549	0.019	0.019	0.457	0.351
SJW	avg	5.562	6.780	0.410	0.590	5.360	5.414
	sd	0.453	0.594	0.085	0.085	0.404	0.316
OC on ***θ***	avg	5.225	7.116	0.463	0.537	4.853	5.709
	sd	0.353	0.529	0.028	0.028	0.603	0.681
HPD	avg	5.225	7.116	0.463	0.537	4.855	5.921
	sd	0.353	0.529	0.028	0.028	0.602	0.471
KL	avg	5.225	7.116	0.304	0.696	5.288	5.439
	sd	0.353	0.529	0.025	0.025	0.476	0.382

This table summaries averages (avg) and standard deviations (sd) of posterior means over 100 replications for OC, AVP, ECR, SJW, OC on ***θ***, HPD and KL, where OC stands for ordering constraints on ***η***, and OC on ***θ*** represents ordering constraints on ***θ***.

For Scenarios (3) and (4) where *η*_1_ = 0.3 and *η*_2_ = 0.7, the correct labels of each replication can be obtained by applying the OC on the posterior samples of ***η***. Averaged posterior means of correctly labelled samples are slightly closer to those of the proposed AVP algorithm than to those of the other algorithms. For Scenario (3), the standard deviations of posterior means of AVP is larger than those of OC; whereas, under Scenario (4), AVP seems to relabel almost all samples back their correct labels.

For Scenarios (1) and (2) where the simulating parameter of ***η*** are set to be equal (*η*_1_ = *η*_2_ = 0.5), the correct labels are unknown. Instead of comparing with the unknown true posterior means, we could compare the similarity between the relabelling algorithms. Among the compared algorithms, ECR and AVP have similar results. The performances of OC on ***θ***, KL and HPD are highly similar to one another, especially in Scenario (4)

### Normal Mixture Models with Known and Unknown Component Weights

In this section, we apply AVP to the unconstrained posterior samples generated from both univariate and multivariate normal mixture models with the number of components to be known and with known and unknown weights. We compare AVP with ECR2 in univariate cases and with DBS in the multivariate cases.

**Univariate cases** For the univariate case, we simulate observation *x*_*i*_ from the normal mixture model, that is,

$$\begin{array}{c} x_{i}∼\sum_{k=1}^{K}\eta_{k}N\left(\mu_{k},V_{k}\right) \end{array}$$ (10)

for *i* = 1, …, *n*, where *μ*_*k*_ and *V*_*k*_ are the mean and the variance of the *k*th component density, respectively. We investigate the simulated model (4.1) studied in [10] with *K* = 3 and *n* = 160. Two scenarios are studied under this model. Scenario (5): ***η*** is known and fixed, and Scenario (6): ***η*** is unknown. The posterior samples of the parameters are generated via the Gibbs sampling scheme suggested by [11], where they assume that the prior distributions are

$$\begin{array}{c} \left(\eta_{1},\ldots ,\eta_{K}\right)∼D\left(1,\ldots ,1\right),\mu_{k}∼N\left(\bar{y},R^{2}\right),\;\text{and}\;,V_{k}∼\Gamma \left(2,R^{2}/200\right),k=1,\ldots ,K, \end{array}$$

where *D*(⋅) is the Dirichlet distribution; Γ(⋅) is the gamma distribution; $\bar{y}$ and *R* are the mean and the range of the data, respectively. Permutation sampler is used in the Gibbs sampling scheme to obtain the 100,000 unconstrained samples (after the burn-in period) of the parameters. Two scenarios are repeated for 100 times. The averages and the standard deviations of posterior means over replications are reported in Table 2. In Scenario (5), ***η*** is assumed to be fixed at true values during the Gibb sampling; hence, the correct labels can be obtained by applying an ordering constraint on ***η***. The differences in averaged posterior means between AVP and ECR2 are small, which are no more than 0.11; however, averaged posterior means of correctly labelling samples are slightly closer to those of AVP than to those of ECR2 (upper part of Table 2). The standard deviations of the posterior means in Table 2 (upper part) show that AVP has better consistence (smaller standard deviations) and is closer to those of correctly labelled samples than ECR2 does.

**Table 2:** The Performances of Algorithms AVP and ECR2 for Univariate Normal Mixture Model under Scenarios (5) and (6).

		Scenario (5): ***η*** are known and fixed								
		*η*_1_	*η*_2_	*η*_3_	*μ*_1_	*μ*_2_	*μ*_3_	*V*_1_	*V*_2_	*V*_3_
OC	avg	0.100	0.250	0.650	-20.009	19.718	20.494	2.004	3.264	2.014
	sd	0.000	0.000	0.000	0.247	0.390	0.172	0.316	0.699	0.294
AVP	avg	0.100	0.255	0.645	-20.009	19.728	20.484	2.004	3.250	2.028
	sd	0.000	0.018	0.018	0.247	0.384	0.171	0.316	0.687	0.294
ECR2	avg	0.100	0.365	0.535	-20.009	19.644	20.568	2.004	3.220	2.058
	sd	0.000	0.059	0.059	0.247	0.465	0.258	0.316	0.703	0.394
		Scenario (6): ***η*** are unknown								
		*η*_1_	*η*_2_	*η*_3_	*μ*_1_	*μ*_2_	*μ*_3_	*V*_1_	*V*_2_	*V*_3_
OC	avg	0.060	0.174	0.766	-0.024	-2.186	20.392	5.421	3.509	2.330
	sd	0.018	0.032	0.049	6.396	6.935	0.151	1.498	0.578	0.300
AVP	avg	0.104	0.139	0.757	-19.961	17.714	20.428	2.906	6.036	2.318
	sd	0.000	0.084	0.084	0.248	0.765	0.196	0.747	1.233	0.564
ECR2	avg	0.104	0.171	0.725	-19.954	17.739	20.396	2.797	6.067	2.396
	sd	0.000	0.103	0.103	0.248	0.827	0.268	0.734	1.379	0.609

The simulating parameter values under these two scenarios are (*η*_1_, *η*_2_, *η*_3_) = (0.1, 0.25, 0.65), (*μ*_1_, *μ*_2_, *μ*_3_) = (−20, 21, 20) and (*V*_1_, *V*_2_, *V*_3_) = (1, 0.5, 3). This table summaries averages (avg) and standard deviations (sd) of posterior means over 100 replications for algorithms OC, AVP and ECR2, where OC stands for ordering constraints on ***η***.

For Scenario (6) where ***η*** is unknown, correct labels are unable to be obtained, leading to the true posterior means are unknown. The results in Table 2 (lower part) show that the simulating parameter values are slightly closer to averaged posterior means of ECR2 than to those of AVP. However, it is noteworthy that true posterior means may not necessarily be close to simulating parameter values because the former could be affected by the setting of prior distributions. The standard deviations of the posterior means show that AVP generally can obtain more consistent estimates in posterior means than ECR2. Putting an ordering constraint on ***η*** (OC) under this scenario could obtain unsatisfactory results, which is informed by its nonsensible estimates for posterior means of *μ*_1_ and *μ*_2_.

**Multivariate cases** To examine the performance and comparison of AVP and DBS in multivariate settings, we simulated data from multivariate normal mixture models. The posterior samples are generated according to [21]. Permutation sampler is adopted in the Gibbs sampling scheme to obtain 100,000 unconstrained samples (after the burn-in period) of the parameters. We study a bivariate normal mixture model with *K* = 4 and *n* = 200, where $x_{i}=\left(x_{i1},x_{i2}\right)∼\sum_{k=1}^{K}\eta_{k}N\left(\mu_{k},\Sigma_{k}\right)$. The prior assumptions are

$$\begin{array}{c} \left(\eta_{1},\ldots ,\eta_{K}\right)∼D\left(1,\ldots ,1\right),\mu_{k}|\Sigma_{k}∼N\left(\zeta_{k},\Sigma_{k}\right),\;\text{and}\;,\Sigma_{k}∼W^{-1}\left(3,\Xi \right),k=1,\ldots ,K, \end{array}$$

where ***ζ***_*k*_ = (*ζ*_*k*1_,*ζ*_*k*2_) and *ζ*_*kj*_ = min_1 ≤ *i* ≤ *n*_{*x*_*ij*_}+*kR*_*j*_/3 with *R*_*j*_ being the range of (*x*_1*j*_, …, *x*_*nj*_), *j* = 1, 2; *W* ^−1^(⋅) is an inverse Wishart distribution and the scale matrix Ξ = *diag*(*δ*_1_, *δ*_2_) with the prior distribution for *δ*_1_ and *δ*_2_ being Γ(2, 36^−1^).

Two scenarios are considered. Scenario (7): ***η*** is known and fixed, and Scenario (8): ***η*** is unknown. Two scenarios are repeated for 100 times and the results are averaged over these replications. Parameter values used to simulate data from Scenario (7) are shown in the first column of Table 3. Notice that this is a challenging case since the true parameter values for one component are extremely close to another. The averaged posterior means in these scenarios are shown in Table 3. As compared with the results from correct labelling (OC), we see that AVP has better performance in the first two component and DBS is better in the fourth component. The standard deviations of posterior means over 100 replications are shown in S1 Table.

**Table 3:** The Performances of Algorithms AVP and DBS for Multivariate Normal Mixture Model under Scenarios (7) and (8).

	Scenario (7): ***η*** are known and fixed			
	simulating parameter values	OC	AVP	DBS
***η***	(0.1, 0.2, 0.3, 0.4)	(0.1, 0.2, 0.3, 0.4)	(0.102, 0.200, 0.300, 0.398)	(0.121, 0.181, 0.300, 0.399)
***μ***	(-3, -3)	(-2.335, -2.605)	(-2.326, -2.601)	(-2.447, -2.696)
	(-3, -3)	(-2.227, -2.651)	(-2.252, -2.665)	(-2.144, -2.577)
	(1, -1)	(0.786, -1.152)	(0.788, -1.151)	(0.790, -1.150)
	(1.1, -0.9)	(1.611, -0.455)	(1.624, -0.447)	(1.636, -0.440)
***V***	$\left(\begin{array}{cc} 1 & 0.5\\ 0.5 & 1 \end{array}\right)$	$\left(\begin{array}{cc} 4.352 & 1.103\\ 1.103 & 3.433 \end{array}\right)$	$\left(\begin{array}{cc} 4.340 & 1.102\\ 1.102 & 3.418 \end{array}\right)$	$\left(\begin{array}{cc} 3.882 & 1.009\\ 1.009 & 3.084 \end{array}\right)$
	$\left(\begin{array}{cc} 1 & 0\\ 0 & 1 \end{array}\right)$	$\left(\begin{array}{cc} 3.060 & 0.945\\ 0.945 & 1.856 \end{array}\right)$	$\left(\begin{array}{cc} 3.071 & 0.946\\ 0.946 & 1.866 \end{array}\right)$	$\left(\begin{array}{cc} 3.534 & 1.040\\ 1.040 & 2.205 \end{array}\right)$
	$\left(\begin{array}{cc} 1 & 0.5\\ 0.5 & 1 \end{array}\right)$	$\left(\begin{array}{cc} 1.231 & 0.284\\ 0.284 & 1.302 \end{array}\right)$	$\left(\begin{array}{cc} 1.229 & 0.283\\ 0.283 & 1.301 \end{array}\right)$	$\left(\begin{array}{cc} 1.229 & 0.282\\ 0.282 & 1.302 \end{array}\right)$
	$\left(\begin{array}{cc} 1 & 0.5\\ 0.5 & 1 \end{array}\right)$	$\left(\begin{array}{cc} 1.263 & 0.234\\ 0.234 & 1.280 \end{array}\right)$	$\left(\begin{array}{cc} 1.267 & 0.235\\ 0.235 & 1.285 \end{array}\right)$	$\left(\begin{array}{cc} 1.261 & 0.234\\ 0.234 & 1.280 \end{array}\right)$
	Scenario (8): ***η*** are unknown			
	simulating parameter values	AVP	DBS	
***η***	(0.25, 0.25, 0.25, 0.25)	(0.250, 0.277, 0.226, 0.247)	(0.250, 0.278, 0.225, 0.247)	
***μ***	(-3, 4)	(-2.850, 3.856)	(-2.934, 3.914)	
	(4.5, -2.5)	(4.606, -2.484)	(4.579, -2.509)	
	(7, -3)	(6.908, -2.598)	(6.947, -2.781)	
	(6.5, 7)	(6.487, 6.892)	(6.560, 7.042)	
***V***	$\left(\begin{array}{cc} 0.5 & -0.25\\ -0.25 & 0.5 \end{array}\right)$	$\left(\begin{array}{cc} 0.966 & -0.219\\ -0.205 & 0.955 \end{array}\right)$	$\left(\begin{array}{cc} 0.933 & -0.233\\ -0.233 & 0.911 \end{array}\right)$	
	$\left(\begin{array}{cc} 0.5 & -0.25\\ -0.25 & 0.5 \end{array}\right)$	$\left(\begin{array}{cc} 1.434 & -0.223\\ -0.223 & 1.620 \end{array}\right)$	$\left(\begin{array}{cc} 1.379 & -0.257\\ -0.257 & 1.485 \end{array}\right)$	
	$\left(\begin{array}{cc} 4 & 2.5\\ 2.5 & 9 \end{array}\right)$	$\left(\begin{array}{cc} 4.984 & 2.721\\ 2.721 & 10.572 \end{array}\right)$	$\left(\begin{array}{cc} 5.044 & 2.750\\ 2.750 & 10.800 \end{array}\right)$	
	$\left(\begin{array}{cc} 4 & 2.5\\ 2.5 & 4 \end{array}\right)$	$\left(\begin{array}{cc} 4.532 & 2.440\\ 2.440 & 4.354 \end{array}\right)$	$\left(\begin{array}{cc} 4.561 & 2.459\\ 2.459 & 4.306 \end{array}\right)$	

This table summaries stimulating parameter values and averages of posterior means over 100 replications for algorithms OC, AVP and DBS, where OC stands for ordering constraints on ***η***.

For Scenario (8) where component weights are unknown, we adopt the bivariate normal mixture model given in [10] for simulating data. In this setting, the averaged posterior means from AVP and DBS are equally close to the true simulating parameter values (lower part of Table 3). The standard deviations of posterior means from AVP seem slightly larger than those from DBS (lower part of S1 Table).

For each relabelling algorithm, we summary its computing time for a relabelling procedure (averaging over 100 replications). Table 4 reports their computation times under scenarios with the same number of components (*K*) and sample size (*n*). Algorithms are run in R 3.1.3 using a personal desktop computer with Inter Core 2 Quad CPU 2.33 GHz. Notice that except the HPD and AVP algorithms, all the algorithms are performed by using label.switching package. Results show that the proposed AVP algorithm can have a long running time when *K* is large. This is because our probabilistic based algorithms requires the computation of *K*! quantities to determine the relabelling permutation per MCMC draw.

**Table 4:** Computation Times in Simulation Studies.

	Algorithm (seconds)
*K* = 2, *n* = 10	AVP (41.6) ECR (81.7) SJW (61.5)
	HPD (284.7) KL (290.2)
*K* = 2, *n* = 100	AVP (65.6) ECR (85.4) SJW (82.3)
	HPD (336.9) KL (332.7)
*K* = 3, *n* = 160	AVP (145.0) ECR2 (230.6)
*K* = 4, *n* = 200	AVP (305.6) DBS (268.6)

The computation times (unit in second) averaging over scenarios having with the same numbers of components (*K*) and sample size (*N*).

## Real Data Analysis

### Model

A common application of mixture model analysis on polytomous response data is the regression extension of the latent class analysis (RLCA) model proposed by Huang and Bandeen-Roche [22]. The basic model of RLCA postulates an underlying categorical latent variable with, say, *K* latent classes, and measured items are assumed independent of one another within each component density. We define **Y**_*i*_ = (*Y*_*i*1_, …, *Y*_*iM*_)^*T*^ to be a set of M polytomous response variables for the *i*th individual, *i* = 1, …, *n*. The *m*th variable, *Y*_*im*_, can take one of values {1, …, *J*_*m*_}, where *J*_*m*_ ≥ 2; the allocation variable, *C*_*i*_, denotes the subpopulation in which the *i*th individual belongs to, and takes a value {1, …, *K*}. The distribution of **Y**_*i*_ can be expressed as the finite mixture density:

$$\mathrm{Pr}\left(Y_{i1}=y_{1},\ldots ,Y_{iM}=y_{M}|x_{i},z_{i}\right)=\sum_{k=1}^{K}\{\eta_{k}\left(x_{i}\right)\prod_{m=1}^{M}\prod_{j=1}^{J_{m}}\pi_{\mathrm{mjk}}^{y_{\mathrm{mj}}}\left(z_{im}\right)\}\text{,}$$ (11)

where *y*_*mj*_ = *I*(*y*_*m*_ = *j*) = 1 if *y*_*m*_ = *j*; 0 otherwise. In addition, this model assumes $\eta_{k}\left(x_{i}\right)=\mathrm{Pr}\left(C_{i}=K|x_{i}\right)$ and $\pi_{mjk}\left(z_{im}\right)=\mathrm{Pr}\left(Y_{im}=j|C_{i}=k,z_{im}\right)$. Covariates $x_{i}={\left(1,x_{i1},\ldots ,x_{ip}\right)}^{T}$ are predictors associated with the allocation variable *C*_*i*_, and $z_{i}=(z_{i1},\ldots ,z_{iM})$ with $z_{im}={\left(z_{im1}\ldots ,z_{imL}\right)}^{T}$ for *m* = 1, …, *M* are covariates built to cause direct influence on response variables. The probabilities $\eta_{k}\left(x_{i}\right)$ and $\pi_{\mathrm{mjk}}\left(z_{im}\right)$ are often implemented assuming the generalized logit link function under the generalize linear model framework [23]:

$$\log[\frac{\eta_{k}\left(x_{i}\right)}{\eta_{K}\left(x_{i}\right)}]=\beta_{0k}+\beta_{1k}x_{i1}+\ldots +\beta_{Pk}x_{iP}$$ (12)

and

$$\log[\frac{\pi_{{\mathrm{mjk}}^{\prime }}\left(z_{im}\right)}{\pi_{{\mathrm{mJ}}_{m}k^{\prime }}\left(z_{im}\right)}]=\gamma_{mjk^{\prime }}+\alpha_{1mj}z_{im1}+\ldots +\alpha_{Lmj}z_{imL}$$ (13)

for *i* = 1, …, *N*;*m* = 1, …, *M*;*j* = 1, …, (*J*_*m*_−1);*k* = 1, …, (*K*−1);*k*′ = 1, …, *K*.

To perform Bayesian analysis on the RLCA model, prior distributions for *β*_*pk*_’s, *γ*_*mjk*_’s and *α*_*lmj*_’s are assumed normal prior distributions with mean 0 and variance 1.5^2^. Parameters *β*_*pk*_’s, *γ*_*mjk*_’s and *α*_*lmj*_’s are sampled in Gibbs sampling approach with acceptance-rejection strategy [24]. The Gibbs sampling scheme for the hierarchical RLCA model are according to Pan and Huang [25]. The following briefly describes the move types:

**Step 1:** For *i* = 1, …, *n*, generate *C*_*i*_ from
  $$\begin{array}{ccc} & & p(S_{ik}|\beta_{01},\ldots ,\beta_{PK},\gamma_{111},\ldots ,\gamma_{MJ_{M}K},\alpha_{111},\ldots ,\alpha_{LMJ_{M}})\\ & = & \frac{\eta_{k}\left(x_{i}\right)\prod_{m=1}^{M}\prod_{j=1}^{J_{m}}\pi_{mjk}^{y_{mj}}\left(z_{im}\right)}{\sum_{k=1}^{K}\eta_{k}\left(x_{i}\right)\prod_{m=1}^{M}\prod_{j=1}^{J_{m}}\pi_{mjk}^{y_{mj}}\left(z_{im}\right)} \end{array}$$ (14)
  with *S*_*ij*_ = *I*(*C*_*i*_ = *j*), and (*S*_*i*1_, …, *S*_*iK*_) can be sampled directly from a multinomial distribution.
**Step 2:** Generate (*β*_01_, …, *β*_*P*(*K*−1)_) from
  $$\begin{array}{ccc} & & p(\beta_{01},\ldots ,\beta_{P(K-1)}|C_{1},\ldots ,C_{n})\\ & \propto & \prod_{i=1}^{n}\eta_{C_{i}}\left(x_{i}\right)\times \prod_{k=1}^{K-1}\prod_{p=0}^{P}p\left(\beta_{pk}\right). \end{array}$$
**Step 3:** Generate (*γ*_111_, …, *γ*_*MJ*_*M*_*K*_) from
  $$\begin{array}{ccc} & & p(\gamma_{111},\ldots ,\gamma_{MJ_{M}K}|\alpha_{111},\ldots ,\alpha_{LMJ_{M}},C_{1},\ldots ,C_{n})\\ & \propto & \prod_{i=1}^{n}\prod_{m=1}^{M}\prod_{j=1}^{J_{m}}\pi_{mjC_{i}}^{y_{mj}}\left(z_{im}\right)\times \prod_{k=1}^{K}\prod_{m=1}^{M}\prod_{j=1}^{J_{m}}p\left(\gamma_{mjk}\right). \end{array}$$
**Step 4:** Generate (*α*_111_, …, *α*_*LMJ*_*M*__) from
  $$\begin{array}{ccc} & & p(\alpha_{111},\ldots ,\alpha_{LMJ_{M}}|\gamma_{111},\ldots ,\gamma_{MJ_{M}K},C_{1},\ldots ,C_{n})\\ & \propto & \prod_{i=1}^{n}\prod_{m=1}^{M}\prod_{j=1}^{J_{m}}\pi_{mjC_{i}}^{y_{mj}}\left(z_{im}\right)\times \prod_{l=1}^{L}\prod_{m=1}^{M}\prod_{j=1}^{J_{m}}p\left(\alpha_{lmj}\right). \end{array}$$
  In addition to the four move types mentioned above, permutation sampling is adopted in the 5th move type.
**Step 5:** Select on the permutation function *v* ^*q*^ for relabelling the current state. Define *θ*_*k*_ = (*β*_0*k*_, …, *β*_*Pk*_, *γ*_11*k*_, …, *γ*_*MJ*_*M*_*k*_) for *k* = 1, …, *K*−1, and $\theta_{K}=\left({\underset{︸}{0,\ldots ,0}}_{P+1},\gamma_{11k},\ldots ,\gamma_{MJ_{M}k}\right)$ for the reference class. Take a new state as *v* ^*q*^(***θ***) = (*θ*_*v*^*q*^(1)_, …, *θ*_*v*^*q*^(*K*)_, *ψ*) and *v* ^*q*^(*S*) = [*S*_*iv*^*q*^(*k*)_]_*i* = 1, …, *n*, *k* = 1, …, *K*_, where *ψ* = (*α*_111_, …, *α*_*LMJ*_*M*__) is the parameter common to all latent classes, and is invariant to permutation function *v* ^*q*^. The new state has to be adjusted to the new reference class in which the *β* coefficients are required to be 0’s.

Adopting the permutation sampling forces the Markov chain quickly to explore all permutation states [15].

### Data

To illustrate the usefulness of the proposed relabelling method, we used data (see S1 File) from two projects: the Multidimensional Psychopathological Study on Schizophrenia (MPSS) project and the Study on Etiological Factors of Schizophrenia (SEFOS) project. The details of study designs are described in detail in Chang et al. [26]. Written informed consent was obtained from all participants after complete description of the studies. These studies (MPSS and SEFOS) were approved by the institutional review boards of the 3 participating hospitals: National Taiwan University Hospital and the university affiliated Taipei City Psychiatric Center and Taoyuan Psychiatric Center. Participants’ consent to the MPSS and SEFOS studies included consent to use their data for other researches. The capacity for consent of patients were assessed by their attending certified psychiatrists to rule out those participants whose psychotic symptoms or mentality were so severe that impair their capacity for consent. All the psychiatric patients who were compulsory hospitalized did not allow to enter our studies. All informed consents were obtained from patients themselves. Proxy consent was prohibited in our studies.

The datasets had been published [27], but not available through any data repositories before. The data had been anonymized prior to access for this study and the age range of participants was from 18–65 years old. The inclusion/exclusion criteria were (i) meeting the DSM-IV diagnostic criteria of schizophrenia, (ii) no history of alcohol and drug abuse, (iii) no neurologic disease, (iv) no mental retardation, (v) no medical illnesses that may significantly impair neurocognitive function.

Briefly, MPSS and SEFOS projects recruited subsided schizophrenia patients (*N* = 225) from three hospitals in Taiwan. The patients are based on the Diagnostic and Statistical Manual of Mental Disorders [28] criteria for schizophrenia. Schizophrenia symptoms used in this study are assessed by the Positive and Negative Syndrome Scale (PANSS) [29, 30]. The PANSS is composed of three subscales and has 30 items (*M* = 30) with positive (seven symptoms, P1–P7), negative (seven symptoms, N1–N7) and general psychopathology (sixteen symptoms, G1–G16). Each item was originally rated on a 7-point scale (1 = absent, 7 = extreme), but the 7-point scale was reduced to the binary scale (*J*_1_ = … = *J*_30_ = 2) (no symptom and having symptom) for easing the sparseness problem of the latent class model. The hierachical RLCA applied here is to explore the underlying subtypes (classes) of schizophrenia based on the PANSS measurement, and to study the relationship between external covariates and obtained patient subtypes. The external covariates used in this study include demographic variables and one neuropsychological variable. Demographic variables are gender, age at recruitment, onset-age of psychotic symptoms, years of education, and occupation (having versus no occupation). The neuropsychological variable is the sensitivity index of the Continuous Performance Test (CPT) [31, 32]. The CPT score is transformed into z-score by comparing to a control group matched for three demographic variables: age, gender and education years [33]. This adjustment was made so that the higher z-score indicates better performance.

The hierarchical RLCA was applied to 30 dichotomized PANSS items. Demographic variables and the z-standardized CPT score were the covariates that were associated with the underlying latent class through Eq (9). Gender and age are identified as covariates incorporated in conditional probabilities in Eq (10). This analysis used the subsample of subjects that without missing values (*N* = 160). The hierarchical RLCA model was fitted through the Gibbs sampling scheme.

### Analysis Results

In this data analysis, we set *K* = 3. We run for 210,000 samples with the first 10,000 samples being the burn-in period. Only every 10 scan is stored to keep independence, and 20,000 samples are recorded for analysis.

**Fig 6:**
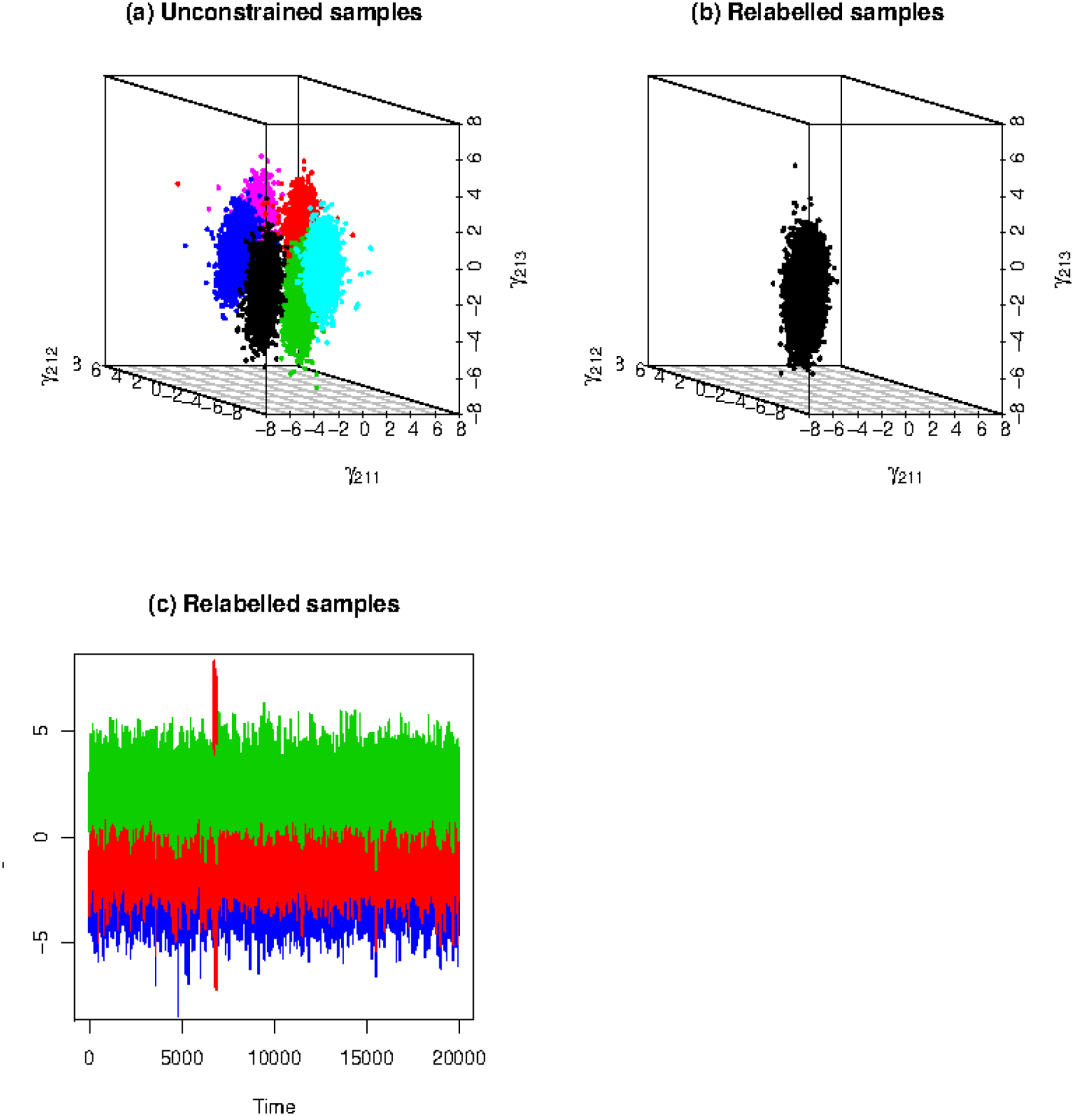
Plot (a) is the 3-dimensional scatter plot of unconstrained sample with (*γ*_211_; *γ*_212_; *γ*_213_). The six colors represent the 3! sets of labels before relabelling. The relabelled samples applied by AVP algorithm are shown in Plot (b). Plot (c) is the trace plots of *γ*_811_, *γ*_812_ and *γ*_813_.

Fig 6a and 6(b) show the unconstrained samples and the relabelled samples after applying the AVP algorithm, respectively, in 3-dimension scatter plots with the dimensions of parameters *γ*_211_, *γ*_212_ and *γ*_213_. Because the schizophrenia syndrome scale data is fitted by a three-component latent class model, Fig 6a with 20,000 samples clearly shows the 3! = 6 clusters in unconstrained posterior samples, distinguished by 6 different colors. Fig 6b shows the relabelled samples after applying the AVP algorithm. The AVP algorithm can identify one out of the 3! sets of unconstrained posterior samples, and relabels the labels of the other 5 sets unconstrained samples to the specific one set. The trace plot of parameters *γ*_811_, *γ*_812_ and *γ*_813_ is shown in the plot of Fig 6c. From these plots, we see that the distributions of parameters are separated well.

After applying the AVP algorithm, the quantities of posterior distributions are summarized in Tables 5 and 6. Table 5 gives the estimation of relationship between subgroups memberships and covariates. The odds ratios (ORs) are the exponential transformation of *β*’s from regression coefficients. The 2.5% and 97.5% quartiles of posterior samples of *β*’s also take the same exponential transformation to obtain the 95% credible interval (CI) of the corresponding ORs. By comparing with the patients from class 3. The characteristics of the other two classes from this analysis are as follows. Patients in class 1 tend to be younger at onset age of psychotic symptoms. Patients in class 2 are more likely to be male, more years of education and better ungraded CPT.

**Table 5:** The relationship between underlying subgroups and covariates from hierarchical LCA.

	group 1 vs. group 3		group 2 vs. group 3	
Variable	OR ^a^	CI ^b^	OR	CI
Male gender	1.08	(0.31, 3.60)	2.80 *	(1.00, 8.22)
Age	0.90 *	(0.82, 0.99)	0.95	(0.88, 1.02)
Age of onset	0.84	(0.68, 1.02)	1.12	(0.94, 1.34)
Years of education	1.73	(0.41, 7.08)	3.80 *	(1.21, 13.20)
Having occupation	1.12	(1.00, 1.28)	1.05	(0.96, 1.17)
Ungraded CPT	1.27	(0.97, 1.69)	1.61 *	(1.25, 2.13)

^a^ OR: odds ratio

^b^ CI: 95% credible interval of OR

* Asterisk is added if value is significantly different from 1, judged by CI not covering 1.

Table 6 contains the direct association between PANSS symptom items and covariates. The ORs are obtained by the exponential transformation of regression coefficients *α*’s. The same exponential transformation is also applied to the 2.5% and 97.5% quantiles of the posterior samples of *α*’s to obtain 95% CI. Males are more likely to have G12 (lack of judgement and insight) symptom than females. The older the age, the higher the probability of having G5 (mannerisms and posturing) symptom and G6 (depression) symptom, but the lower the probability of having N4 (passive/apathetic social withdrawal) symptom.

**Table 6:** The association between the PANSS symptoms’ probability and covariates from hierarchical RLCA.

		Male Gender		Age	
	Variable	OR ^a^	CI ^b^	OR	CI
P1	Delusis	1.20	(0.55, 2.63)	1.01	(0.97, 1.06)
P2	Conceptual disorganization	0.96	(0.39, 2.33)	1.04	(0.99, 1.09)
P3	Hallucinatory behavior	1.13	(0.52, 2.49)	1.03	(0.99, 1.08)
P4	Excitement	1.11	(0.44, 2.82)	1.03	(0.98, 1.09)
P5	Grandiosity	1.57	(0.63, 4.00)	1.01	(0.96, 1.06)
P6	Suspiciousness/persecution	1.95	(0.87, 4.43)	1.01	(0.96, 1.05)
P7	Hostility	1.24	(0.49, 3.15)	1.01	(0.96, 1.06)
N1	Blunted affect	0.42	(0.13, 1.14)	0.98	(0.93, 1.03)
N2	Emotional withdrawal	1.20	(0.44, 3.18)	0.98	(0.93, 1.03)
N3	Poor rapport	0.59	(0.20, 1.57)	1.03	(0.97, 1.08)
N4	Passive/apathetic social withdrawal	1.34	(0.50, 3.50)	0.93 *	(0.89, 0.98)
N5	Difficulty in abstract thinking	0.85	(0.37, 1.92)	1.02	(0.97, 1.06)
N6	Lack of spontaneity/flow of conversation	0.81	(0.33, 1.90)	1.03	(0.98, 1.08)
N7	Stereotyped thinking	1.84	(0.79, 4.29)	1.03	(0.98, 1.08)
G1	Somatic concern	0.90	(0.43, 1.86)	1.00	(0.96, 1.04)
G2	Anxiety	1.04	(0.49, 2.18)	1.01	(0.97, 1.06)
G3	Guilt fellings	0.42	(0.17, 1.02)	1.00	(0.95, 1.04)
G4	Tension	0.56	(0.24, 1.27)	0.99	(0.95, 1.04)
G5	Mannerisms and posturing	1.27	(0.42, 4.00)	1.08 *	(1.02, 1.16)
G6	Depression	1.08	(0.49, 2.37)	1.06 *	(1.01, 1.12)
G7	Motor retardation	0.68	(0.27, 1.66)	1.04	(0.99, 1.10)
G8	Uncooperativeness	1.15	(0.44, 3.04)	1.03	(0.98, 1.09)
G9	Unusual thought content	0.96	(0.41, 2.21)	1.03	(0.98, 1.08)
G10	Disorientation	0.39	(0.14, 1.03)	1.00	(0.95, 1.06)
G11	Poor attention	1.16	(0.46, 2.89)	1.01	(0.96, 1.06)
G12	Lack of judgement and insight	2.58 *	(1.16, 5.85)	0.98	(0.93, 1.02)
G13	Disturbance of volition	1.04	(0.48, 2.25)	1.02	(0.98, 1.07)
G14	Poor impulse control	0.76	(0.31, 1.85)	1.04	(0.99, 1.10)
G15	Preoccupation	0.73	(0.26, 1.98)	1.01	(0.96, 1.07)
G16	Active social avoidance	0.68	(0.31, 1.47)	1.01	(0.97, 1.06)

^a^ OR: odds ratio

^b^ CI: 95% credible interval of OR

* Asterisk is added if value is significantly different from 1, judged by CI not covering 1.

## Conclusion

The proposed AVP algorithm has the following features. (i) AVP is attributed to probabilistic approach, which prevents over-corrected results compared with deterministic methods. (ii) AVP seems to perform reasonably well with the limiting settings in our simulation studies. (iii) The computation time of AVP depends on the dimension of allocation variables **S** (i.e., the number of observations (*n*) and the number of components (*K*) in the mixture model), but not on the complexity of the density function of mixture models. That is, even when data is drawn from a complicated mixture model, the computational cost for AVP holds the same as that from the models where have the same numbers of observations and components. (iv) AVP can have a long computation time when *K* is large, since a probabilistic based algorithm requires the computation of *K*! quantities to find the optimal permutation per MCMC draw.

## Supporting Information

S1 Table The Performances of the AVP and DBS Algorithms for Multivariate Normal Mixture Model under Scenarios (7) and (8). This table summaries standard deviations of posterior means over 100 replications for algorithms OC, AVP and DBS, where OC stands for ordering constraints on ***η***.
(PDF)

S1 File Raw data of the study sample. This dataset contains 30 outcome variables and 6 explanatory variables. The variables are summarised as follows and variable names are shown parenthetically. The 30 outcome variables are seven positive symptoms (P1–P7), seven negative symptoms (N1–N7) and sixteen general psychopathology symptoms (G1–G16) with binary response with 0 = no symptom and 1 = having symptom. The 6 explanatory variables are gender (Male_gender) with 0 = female and 1 = male, age at recruitment (Age), onset-age of psychotic symptoms (Age_of_onset), years of education (Year_of_education), occupation (Having_occupation) with 0 = no occupation and 1 = having occupation and CPT score (Ungraded_CPT).
(CSV)
