# Supplementary material for: Allocation Variable-Based Probabilistic Algorithm to Deal with Label Switching Problem in Bayesian Mixture Models
Source: PLoS One. 2015 Oct 12;10(10):e0138899. doi: 10.1371/journal.pone.0138899 (PMC4601799; doi:10.1371/journal.pone.0138899)
Supplement: S1 Table — This table summaries standard deviations of posterior means over 100 replications for algorithms OC, AVP and DBS, where OC stands for ordering constraints on η. (PDF) [file pone.0138899.s001.pdf]

	Scenario (7): $\boldsymbol{\eta}$ are known and fixed		
	OC	AVP	DBS
$\boldsymbol{\eta}$	(0, 0, 0, 0)	(0.010, 0.008, 0.001, 0.012)	(0.008, 0.007, 0.003, 0.008)
$\boldsymbol{\mu}$	(0.201, 0.137)	(0.227, 0.148)	(0.205, 0.135)
	(0.206, 0.119)	(0.177, 0.119)	(0.182, 0.127)
	(0.107, 0.092)	(0.108, 0.092)	(0.109, 0.092)
	(0.223, 0.163)	(0.174, 0.142)	(0.133, 0.122)
$\boldsymbol{V}$	$\begin{pmatrix}0.223 & 0.197\\0.197 & 0.170\end{pmatrix}$	$\begin{pmatrix}0.238 & 0.199\\0.199 & 0.169\end{pmatrix}$	$\begin{pmatrix}0.203 & 0.194\\0.194 & 0.156\end{pmatrix}$
	$\begin{pmatrix}0.204 & 0.152\\0.152 & 0.135\end{pmatrix}$	$\begin{pmatrix}0.207 & 0.153\\0.153 & 0.151\end{pmatrix}$	$\begin{pmatrix}0.201 & 0.153\\0.153 & 0.132\end{pmatrix}$
	$\begin{pmatrix}0.074 & 0.061\\0.061 & 0.078\end{pmatrix}$	$\begin{pmatrix}0.071 & 0.060\\0.060 & 0.077\end{pmatrix}$	$\begin{pmatrix}0.072 & 0.060\\0.060 & 0.078\end{pmatrix}$
	$\begin{pmatrix}0.132 & 0.095\\0.095 & 0.115\end{pmatrix}$	$\begin{pmatrix}0.152 & 0.095\\0.095 & 0.127\end{pmatrix}$	$\begin{pmatrix}0.125 & 0.094\\0.094 & 0.114\end{pmatrix}$
	Scenario (8): $\boldsymbol{\eta}$ are unknown		
	AVP	DBS	
$\boldsymbol{\eta}$	(0.002, 0.022, 0.023, 0.005)	(0.001, 0.019, 0.020, 0.005)	
$\boldsymbol{\mu}$	(0.419, 0.296)	(0.109, 0.105)	
	(0.326, 0.272)	(0.218, 0.171)	
	(0.381, 0.781)	(0.306, 0.645)	
	(0.486, 0.470)	(0.261, 0.260)	
$\boldsymbol{V}$	$\begin{pmatrix}0.210 & 0.138\\0.138 & 0.260\end{pmatrix}$	$\begin{pmatrix}0.102 & 0.081\\0.081 & 0.088\end{pmatrix}$	
	$\begin{pmatrix}1.147 & 0.231\\0.231 & 2.775\end{pmatrix}$	$\begin{pmatrix}0.982 & 0.240\\0.240 & 2.064\end{pmatrix}$	
	$\begin{pmatrix}2.312 & 1.152\\1.152 & 3.117\end{pmatrix}$	$\begin{pmatrix}2.319 & 1.148\\1.148 & 2.977\end{pmatrix}$	
	$\begin{pmatrix}0.753 & 0.644\\0.644 & 0.813\end{pmatrix}$	$\begin{pmatrix}0.729 & 0.626\\0.626 & 0.726\end{pmatrix}$	
